# Tumor and Microenvironment Modification during Progression of Murine Orthotopic Bladder Cancer

**DOI:** 10.1155/2011/865684

**Published:** 2011-10-13

**Authors:** Sin Mun Tham, Kee Hui Ng, Sim Hwee Pook, Kesavan Esuvaranathan, Ratha Mahendran

**Affiliations:** Department of Surgery, Yong Loo Lin School of Medicine, National University of Singapore, 1E Kent Ridge Road, NUHS Tower Block, Level 8, Singapore 119228

## Abstract

The aim of this study was to monitor changes in the expression of immune-related genes in the bladder after tumor implantation. Mice were orthotopically implanted with MB49-PSA cells (C57BL/6 mice) on day 1 and terminated on days 7, 14, 21, and 28. Another mouse model (MBT-2/C3H mice) was examined at day 7. Gene expression analysis was performed using a TaqMan Low Density Mouse Immune Panel (Applied Biosystems, USA) on RNA extracted from the bladders. Selected genes were reconfirmed by real-time PCR analysis and RT-PCR on the mRNA from other animals. Immune suppressive (IL13, IL1**β**, PTGS2, NOS2, IL10, CTLA4, and CCL22) and immune stimulatory genes (CSF2, GZMB, IFN**γ**, CXCL10, TNF**α**, CD80, IL12a, and IL6) and AGTR2 were increased by day 7. By day 28, IL10, CCL2, CCL5, CXCL11, CTLA4, GZMB, IFN**γ**, CSF2, and IL6 were significantly increased. Therapeutic strategies involving TH1 induction and TH2 dampening may improve responses to immunotherapy.

## 1. Introduction

Bladder cancer is the 7th most common cancer worldwide. Though bladder cancer is not usually life threatening, it is prone to recurrences which may progress to invasive cancer. Transurethral resection of the bladder tumor (TURBT) followed by Mycobacterium bovis, Bacillus Calmette Guerin (BCG) immunotherapy reduces the incidence of recurrence, but some 30–50% of patients do not respond to therapy [1, 2]. Recurrences are attributed to remnant tumor cells missed during surgery as a second surgical procedure prior to BCG therapy improves the response to therapy [1]. The immune response induced by BCG immunotherapy may inadvertently ensure the survival of less immunogenic remnant tumor cells which could give rise to recurrence and progression. The increased incidence of progression in patients who fail BCG immunotherapy gives some credence to this latter possibility [1]. 

Tumor immune editing is a dynamic process that has 2 important participants the tumor cells and the immune cells. Their interaction determines whether tumor regression or growth occurs. Orthotopic murine models of bladder cancer generated by implanting syngenic cell lines have been used to evaluate response to gene therapy. One such model, MB49 cells implanted in C57BL/6 mice, has been shown to be fairly similar to human bladder cancers [3]. Several cytokine genes have been evaluated for their ability to induce tumor regression in this model. Intravesical delivery of IL2 cured 40% of mice [4], IFN*γ* cured 50–80% of mice based on the amount of IFN*γ* secreting retrovirus delivered [5], TNF*α* cured 67% of mice [6], IFN*α* singly and/or with GMCSF cured 20–50% of mice [7, 8], adenovirus delivery of IL12 cured 88% of mice [9], and AdCD40 L delivery cured 60% of mice [10]. Tumor cells expressing IL12 and IL18 were completely rejected in mice [11]. The poor response to intravesical therapy has been attributed to the inadequacies of the gene delivery systems whether viral [12] or nonviral [13, 14]. However, MB49 cells were shown by Yang and Lattime [15] and Halak et al. [16] to induce the expression of IL10 in the bladder. IL10 is an immunosuppressive gene and its expression may have contributed to the poor response. Besides IL10, there may be other immunosuppressive mechanisms involved in the interplay between the immune system and the tumor cells.

The aim of this study therefore was to characterize the changes in the bladder environment after orthotopic tumor cell implantation. In general, tumors implanted in the bladder if not treated result in death after about 4–6 weeks. Some mice may show spontaneous cures in this time period. To understand the events that occur during tumor growth in the bladder environment, we chose to evaluate gene expression changes soon after tumor cell implantation (7th day) and at a later stage (28th day). Several immunosuppressive genes were induced after tumor implantation, and these represent possible new targets for therapy. A few mice were also examined 14 and 21 days after tumor implantation. Our results indicate that the TH1/TH2 balance in the tumor environment varies with time and may influence the success of any therapy.

## 2. Methods

### 2.1. Tumor Implantation

All animal work adhered to the National University of Singapore, Institutional Animal Care and use Committee (IACUC), guidelines on animal use and handling. Four to six-week-old female C57BL/6 mice were orthotopically implanted with MB49-PSA (prostate-specific antigen secreting MB49 cells) [8] using poly-L-lysine (PLL) as described by Ninalga et al. [17]. Mice were anesthetized with 75 mg ketamine/kg and 1 mg medetomedine/kg of animal weight (0.1 mL/10 g mice body weight) prior to implantation. MB49 cells were originally obtained from Prof T Ratliff, Purdue University. Briefly, mice were given a bladder instillation of 0.1 mL of sterile 0.01% PLL (Sigma-Aldrich, St. Louis, Mo, USA) for 20 mins prior to instillation of the tumor cells (1 × 10^6^ cells/mL) for 2 hours. Control mice were treated with PLL but not implanted with tumor cells. PSA levels in urine were monitored using a free-PSA chemiluminescence ELISA kit (Autobio Diagnostics, Zhengzhou, China) and normalized against creatinine levels using a kit (Wako Pure Chemicals Industries, Osaka, Japan). Mice were killed on days 7 and 28 and RNA isolated from the bladders. For gene expression studies, 10 mice were used per group. To determine gene expression changes with time, at least 3 mice were sacrificed on days 7, 14, 21, and 28. For flow cytometry studies, 6 mice were sacrificed on day 28 and tissues were pooled from 2 mice for analysis.

Similarly, four-to-six-week-old female C3H/HeJ mice were orthotopically implanted with MBT2-PSA (prostate-specific antigen secreting MBT2 cells) while control C3H/HeJ mice were treated with PLL only. After 7 days, 4 mice from each group were sacrificed for gene expression analysis.

### 2.2. RNA Isolation and Real-Time LDA Panel

Frozen bladders and lymph nodes were soaked overnight in RNA*later*-ICE (Ambion, Austin, Tex, USA) at −20°C before they were homogenized in TRIzol Reagent (Invitrogen, Carlsbad, Calif, USA). CDNA was reverse transcribed from total RNA (10 *μ*g per sample) with random primers using the High-Capacity cDNA Reverse Transcription Kit with RNase Inhibitor (Applied Biosystems, Foster City, Calif, USA). MRNA expression was analyzed using a TaqMan Low Density Mouse Immune Panel (Applied Biosystems) containing a panel of 96 immune-related genes (cytokines, chemokines, growth factors, immune regulators, apoptosis markers, ischemia markers, tissue-specific markers, and others including classic and endogenous controls) as described by Cai et al. [18]. Detection and analysis was performed on the ABI PRISM 7900HT Sequence Detection System with ABI 7900HT SDS Software Version 2.2.1 with the following parameters: 2 mins at 50°C, 10 mins at 94.5°C, and 40 cycles of 30 s at 97°C for denaturation and 1 min at 59.7°C for transcription. Differential gene expression profiling was performed using the comparative *C_T_* method of relative quantitation. All samples were loaded in triplicate, and results with *C_T_* SD ≥ 0.3 were discarded. The lowest limit of detection is *C_T_* = 32 thus any gene with a *C_T_* value beyond 32 is considered not detectable. Only genes displaying at least a 2-fold difference in expression level relative to control were considered to be upregulated. Gene expression was confirmed using real-time PCR analysis for a select group of genes.

### 2.3. Real-Time PCR Analysis and RT-PCR

Real-time PCR reactions for single genes were performed using 100 ng of reverse transcribed RNA, TaqMan universal PCR master mix and pre formulated 20x TaqMan gene expression assay in a 96-well PCR plate. The genes and their TaqMan gene expression assay numbers are Actb Mm00607939_s1; AGTR2 Mm00431727_g1; CSF2 Mm00438328_m1; CTLA4 Mm00486849_m1; CXCR3 Mm00438259_m1; GAPDH Mm99999915_g1; GZMB Mm00442834_m1; IFN*γ* Mm00801778_m1; IL10 Mm00439616_m1; PTGS2 Mm00478374_m1; TGFb1 Mm00441724_m1; TNF*α* Mm00443258_m1. Detection and analysis was performed on the ABI 7500 Real-Time PCR System with the ABI 7500 System Sequence Detection Software Version 1.3.1. PCR was performed for forty cycles with the following parameters: 2 mins at 50°C, 10 mins at 95°C, and for each cycle 15 s at 95°C for denaturation and 1 min at 60°C for transcription. All samples were measured in triplicates and normalized with beta actin. The lowest limit of detection is *C_T_* = 35.

Semiquantitative PCR was performed as described before [19] for 35 cycles. The PCR products were separated on agarose gels, and band intensities were quantified with Gene Tools analysis software (SynGene, Cambridge, England) and normalized against GAPDH or beta actin and expressed as relative quantitation (RQ).  Table 1 lists the genes, primer sequences, annealing temperature, and the size of the PCR products.

### 2.4. Flow Cytometry Analysis

Bladders were harvested, placed in RPMI-1640, and cut into smaller pieces before digestion with collagenase (Sigma-Aldrich) for 30 mins at 37°C. The suspension was filtered through a 70 *μ*m cell strainer (BD, Franklin Lakes, NJ, USA) and centrifuged at 10.4 g for 5 mins at 4°C. The samples were incubated in RBC lysis buffer (150 mM NH_4_Cl, 10 mM KHCO_3_, and 0.1 mM EDTA) for 5 mins at room temperature and rinsed twice in cold PBS. The number of cells in each sample was enumerated with a haemocytometer. Pooled samples were resuspended in buffer containing 1% (w/v) BSA and 0.01% sodium azide in 1xPBS. Samples were assessed for T cells using fluorescein isothiocyanate- (FITC-) conjugated anti-CD3, phycoerythrin- (PE-) conjugated anti-CD4 and anti-CD8a. Macrophages and NK cells were detected with anti-Mac3-FITC and anti-Pan NK-PE. All antibodies were from BD. Flow cytometry was performed on a CyAn_ADP_ (Dako Cytomation, Sweden). The data obtained was analyzed with the Summit software. 

### 2.5. Statistical Analysis

The SPSS statistical package (SPSS, Ill, USA) was used to analyze statistical significance. The *t*-test for equality of means was used to analyze the gene expressions of two groups of animals (normal and tumor bearing). To analyze variance between the gene expressions in the different groups of animals, one-way ANOVA (Bonferroni) was used. Bivariate correlation was used to analyze correlation between two arrays. A *P* value of ≤0.05 was deemed to be significant.

## 3. Results

### 3.1. Tumor Growth and Gene Expression

**Figure 1:**
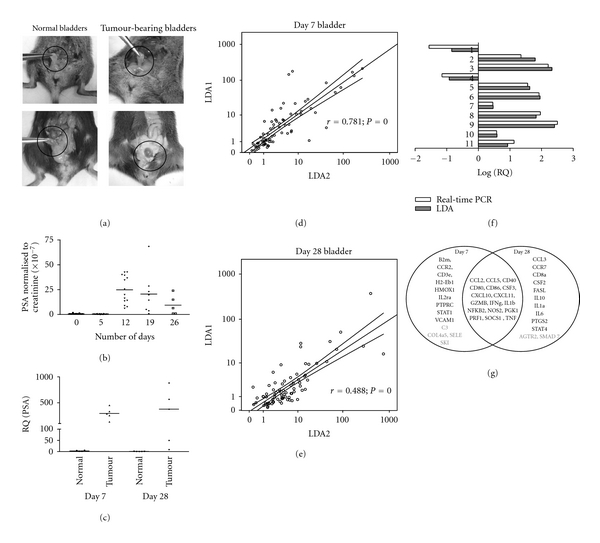
An orthotopic murine model of bladder cancer was established and gene expression analysis performed on day 7 and day 28. (a) Neovascularization was apparent in the bladders 7 days after tumor implantation (upper panel), and after 28 days, the tumor-bearing bladder appeared highly vascular and larger than normal bladders (lower panel). (b) Tumor growth was monitored by measuring urinary PSA which was normalized with creatinine. (c) To confirm the presence of tumors, PSA gene expression in the bladder was analyzed by real-time PCR when the mice were culled at day 7 and day 28. Gene expression in the bladder was analyzed using the LDA arrays and 2 individual mice bladders. The scatter diagrams show the relationship between the RQ values of the 2 arrays of the 2 mice for the (d) day 7 and (e) day 28 bladders. The axes of the scatter diagrams are in the logarithmic scale. The middle line is the best-fit line and the two lines flanking represent the 95% confidence interval. *r* is the Pearson's correlation, and *p* is the significance level. To reconfirm the array data several genes were reanalyzed by real-time PCR using the same murine samples. (f) The log (RQ) shows the same profile for all the genes evaluated. The 11 genes are (1) AGTR2; (2) CTLA4; (3) IFN*γ*; analyzed on RNA from mouse sample one and (4) AGTR2; (5) CSF2; (6) CTLA4; (7) CXCR3; (8) GZMB; (9) IFN*γ*; (10) PTGS2 and (11) TNF*α* analyzed on RNA from mouse sample 2. (g) A summary of genes with increased or decreased expression on days 7 and 28 after tumor implantation.

The murine bladder cancer cell line MB49 was modified to secrete PSA so that PSA levels could be used as a surrogate marker for the presence of tumors [8]. MB49-PSA, like the parental MB49, is fairly immunogenic, and some mice were spontaneously cured (35%) after tumor implantation. MB49 cells express the HY antigen as they were originally isolated from a male mouse [20] as well as BLCA-4, PSCA, and STEAP [21] and consequently are immunogenic, and some mice may become cured without any therapy [6, 7]. The tumor implantation technique used has a 100% implantation efficiency [17], and this was confirmed by measuring PSA secretion in the urine. The growth of the tumors in the bladder was also monitored by measuring urinary PSA levels.  Figure 1(a), top right panel shows tumor bearing bladders harvested at day 7, when the tumors were small and at day 28 (bottom right panel) when the tumors were large. In all mice, an initial increase in urinary PSA secretion was observed at day 11 but later as some mice were cured, PSA secretion decreased, Figure 1(b). 

To confirm the presence of tumors at the point of termination of the study, PSA mRNA levels were determined by real-time PCR analysis, Figure 1(c). This is especially important in mice with low PSA protein levels in urine as real-time PCR analysis is a more sensitive method to confirm the presence of tumors. RNA isolated from samples with confirmed tumors was used to probe a real-time gene expression array of cytokine/chemokine genes. For each time point, two independent samples were analyzed on the arrays. The correlation between the arrays is shown in Figures 1(d) and 1(e). The data from the day 7 arrays showed greater homology than those from the day 28 arrays indicating the greater diversity of the evolving tumor and the tumor microenvironment. To validate the array data, 9 genes were selected to be reconfirmed by real-time PCR using the same mice samples that were used on the array. The LDA data was comparable with the data obtained from real-time analysis, Figure 1(f) confirming the validity of the array data. 

The genes up- and downregulated on both arrays are summarized in Figure 1(g). Seventeen genes were upregulated in both day 7 and day 28 bladders as shown in Figure 1(g). At Day 7, proteins associated with MHC class I and II receptors (*β*2M, CD3e, H2-EB1, HMOX1, and PTPRC) and monocytes and dendritic cells (CD40, CD80, and CD86) chemokine and cytokine genes (CCL2, CCL5, CSF3, CXCL10, CXCL11, GZMB, IFN*γ*, IL1b, NOS2, PGK1, PRF1, SOCS1, and TNF) and receptors (IL2ra) attachment and trafficking proteins (CCR2, VCAM1) and the transcription factors that regulate their expression (STAT1, NFKB2) were upregulated. Genes upregulated uniquely by day 28 include CCL3, CCR7, CD8a, CSF2, FASL, IL10, IL1a, IL6, PTGS2, and STAT4. IL10, PTGS2, and FASL are known to suppress the immune response and SMAD7 which inhibit TGF*β*, and bone morphogenetic protein expression was downregulated which would lead to an environment favoring tumor growth. But there were increased CSF2, CCL3, and IL6 which would recruit immune cells as well as increased CD8a expression. Thus, the tumor environment is a dynamic one with infiltrating immune cells trying to destroy the tumor in an environment that can be inhibitory to their activation and/or action. 

### 3.2. Validating Gene Expression Changes and Immune Cell Infiltration in Tumor Bearing Mice

To validate the array data, the expression of selected genes was assayed in other mice. This included 17 of the differentially expressed genes from Figure 1(g) and 12 that were determined to be similar to controls. The expression of these genes in MB49 cells was also examined.  Table 2 summarizes the results of the analyses which were performed using either real-time analysis or semiquantitative PCR.

Several of the genes, whose expression was increased in the bladder at day 7, were found to be expressed by MB49 cells, such as CSF2, PTGS2, CXCL10, TGF*β*1, CCL2, and CCL5. The increased expression of IFN*γ* in the bladder at day 7 is probably due to CXCL10 being secreted by the tumor cells (Table 2), but MB49 itself does not express IFN*γ*. 

Increased expression of CD80, GZMB, and CTLA4 is probably due to immune cell infiltration of the bladder at day 7. By day 28, GZMB and CTLA4 were still significantly increased and CD80 was elevated compared to control animals. Immune cells in the bladder at day 28 were analyzed using flow cytometry, Table 3. There was increased CD3^+^CD8^+^ in the bladder at day 28 which was consistent with the increased CD8a expression noted on the array. B cells were significantly increased in the bladder at day 28, Table 3. The presence of CTLA4 may explain the lack of tumor reduction despite infiltrating immune cells. CTLA4 binds to CD80 on antigen-presenting cells and blocks the activation of T cells. The transcription factors TBET, GATA3, and FOXP3 regulate TH1, TH2, and Treg development. GATA3 was low in the bladder of tumor-bearing mice at day 7, and FOXP3 was increased indicating the presence of suppressor cells.

Using PSA expression as a measure of tumor size, we segregated the mice as cured and mice bearing small tumors (mean PSA expression 73.03 ± 69.92), medium tumors (mean PSA expression 311.2 ± 45.85), and large tumors (mean PSA expression 1602 ± 1914) for the day 28 samples, Table 4. There were a clear and significant decrease in AGTR2 expression and increase in CTLA4 and GMCSF expression with increasing PSA levels. Further, it appeared that the large tumors expressed high levels of TH1 cytokines like IFN*γ* and even TNF*α*. Thus, there must be immune-suppressive factors blocking tumor eradication.

As a further test of the robustness of the data obtained, another mouse tumor model, C3H mice bearing MBT-2 tumors, was also evaluated. About 36.8% (7/19) of the genes, namely, CSF2, TNF*α*, CCL22, CXCL10, IL13, IL1b, and IFN*γ* were significantly upregulated in both mice models by day 7. 

### 3.3. The Immune Environment Shapes the Tumor

**Figure 2:**
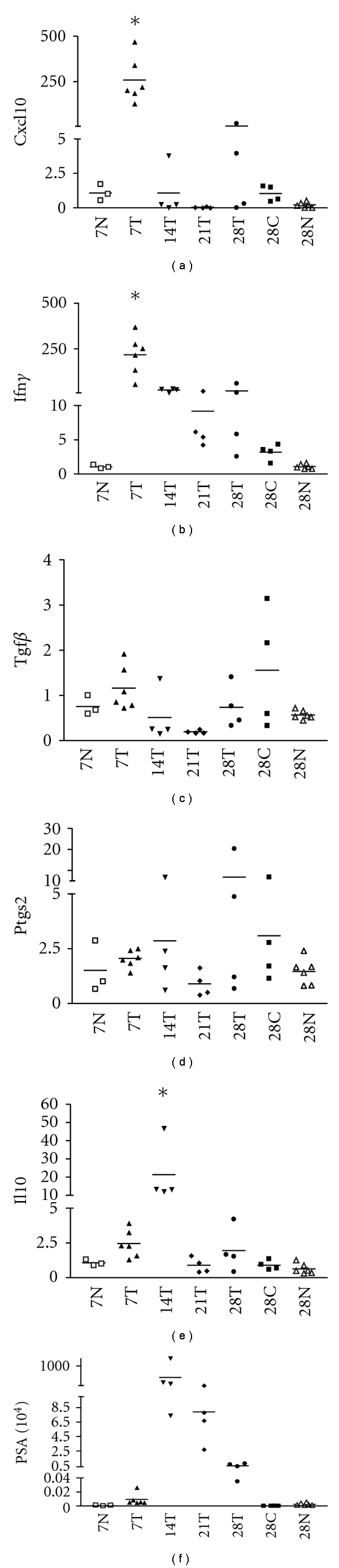
Monitoring gene expression changes in the bladder days after tumor implantation. The genes analyzed were (a) CXCL10; (b) IFNg; (c) TGFb; (d) PTGS2; (e) IL10; (f) PSA. Samples obtained from normal healthy bladders are labeled N, those from tumor bearing mice are labeled T, and samples obtained from cured mice are labeled C. Each point represents one murine bladder sample. * indicates a significant difference (*P* < 0.05) with respect to all the other groups.

To monitor the change in gene expression with time, bladders were also harvested at day 14 and 21 after tumor cell implantation and examined for the expression of IFN*γ*, CXCL10, TGF*β*, IL10, and PTGS2. IL10 and IFN*γ* were chosen as these are not produced by the tumor cell line and their presence denotes expression by immune or other urothelial cells. Further, both these cytokines were upregulated in the two tumor models that were evaluated. IFN*γ* decreased with time as did CXCL10, but not as dramatically as CXCL10. This was not a result of tumor reduction but of the process of selection for a less immunogenic cell type as PSA levels did not decrease as dramatically (Figures 2(a) to 2(d)). Analysis of day 14 and day 21 samples showed that the decrease in CXCL10 from days 7 to 14 was followed by a concomitant increase in IL10 over the same period. Thus, while the tumor changes the environment in the bladder, as shown by the increase in IFN*γ* and IL10, it too is changed by the activity of the immune cells recruited to the bladder which selectively destroy the immunogenic cells and thus encourage the survival of less immunogenic cells. 

## 4. Discussion

A comparison of the gene expression patterns in tumor-bearing animals revealed the variability of gene expression, as there were only a 55% homology at day 7 and a 41.6% homology at day 28 in gene expression between mice. This indicates the heterogeneous nature of the tumor cells that were implanted. As tumors grew in the bladder, there were both sculpting of the tumor cells by immune cells as well as suppression of the immune cells infiltrating the tumor by the tumors themselves.

**Figure 3:**
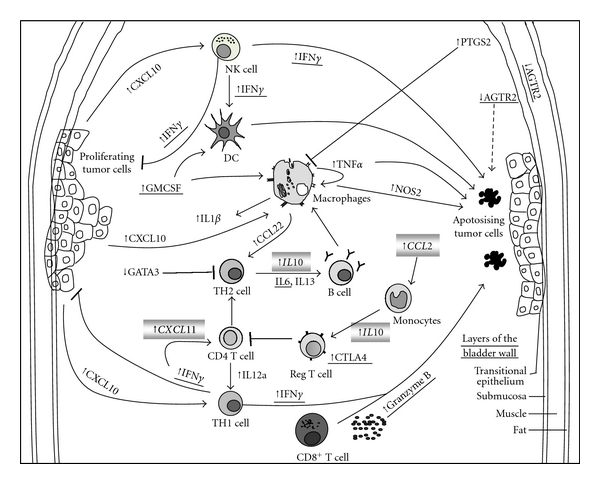
A schematic diagram that summarizes the gene expression changes and immune cells identified in bladders after tumor implantation. Proliferating tumor cells increase the expression of GMCSF and CXCL10 which in turn recruit immune cells such as natural killer (NK) cells, dendritic cells (DC), macrophages, and T cells to the bladder. Increased expression and production of IFN*γ*, TNF*α*, IL12a, NOS2, and GZMB by the immune cells would inhibit tumor proliferation and induce apoptosis. Transcription factor GATA3 was downregulated, suppressing the production of TH2 T cells. CTLA4 binds to CD80 and inhibits T-cell activation. Upregulation of PTGS2 in either tumor or immune cells or both would have an inhibitory effect on immune cells. Downregulation of AGTR2 could have occurred in the tumor cells, normal bladder epithelia, or submucosa. The dotted arrow indicates that AGTR2 may cause apoptosis in tumor cells. Genes that are underlined were present at both day 7 and day 28. Genes in the shaded box were found only at day 28. Genes whose expression was upregulated are indicated by arrows pointing up, and those that were downregulated are indicated by arrows pointing down.

By day 7, IFN*γ*, CSF2, CXCL10, GZMB, PTGS2, TNF*α*, CD80, CCL22, IL12a, IL13, IL1b, IL6, NOS2, and CTLA4 were significantly upregulated in the tumor-bearing mice bladders and AGTR2 and GATA3 were downregulated. These genes could lead to active immune cell recruitment (CSF2, CXCl10, and IFN*γ*) and modulation of immune cell activation (IL12a, IFN*γ*, CSF2, CD80, GZMB, NOS2, and CTLA4), and the generation of an inflammatory environment (IL6 and IL1b). Based on the genes identified granulocytes, NK cells and T cells would be attracted by CSF2, CXCL10, and CCL22 to the bladder. As a consequence of the presence of NK and T cells, there would be increased expression of GZMB and IFN*γ* Figure 3. TNF*α* could induce direct cytotoxic effects on tumor cells [6]. The inhibitory activity of IL13, IL1b, PTGS2, NOS2, and CTLA4 [22–26] on the immune response would enhance tumor growth. CCL22 has been linked to the recruitment of suppressor T cells [27], but it also recruits other immune cells which may reduce tumor growth in the absence of Tregs. Similarly, IL13 has been reported to be both pro- and antitumor [22, 28]. By day 28, IL10, CCL2, CCL5, and CXCL11 which were slightly increased at day 7 were significantly increased in the bladder. Also CTLA4, GZMB, IFN*γ*, TNF*α*, and IL6 remained significantly increased in the bladder, Figure 3. When gene expression was analyzed with respect to tumor sizes, both immunosuppressive (CTLA4) and immune stimulatory (IFN*γ* and TNF*α*) genes were increased with increasing tumor size.

Increased CD8 T cells and B cells were found in the bladder at day 28. In human urothelial cancer tissue, increased tumor infiltrating CD8 T cells correlates with better disease-free survival [29]. However, we have previously found that immune cells recruited to the bladder are usually located at the periphery of the tumor mass [30] and may thus be ineffective in the tumor environment. This could be caused by the immunosuppressive genes identified in this study such as CTLA4. The MB49 cells express several tumor antigens as do human bladder tumors [31]. It is likely that immunosuppressive molecules induced by the tumors may prevent the activation of an adequate immune response against the tumor antigens. In support of this view, bladder cancer patients receiving anti-CTLA4 therapy showed an increase in the ratio of effector to regulatory T cells as well as tumor antigen-specific CD4 T cells [24].

AGTR2 was consistently downregulated in all tumor bearing samples. This receptor downregulates the stimulatory effect of EGF on the growth of prostate cancer cells [32]. AGTR2 acts with TIMP3 to block angiogenesis [33], but in AGTR2 knockout mice there was impaired induction of peripheral angiogenesis [34]. Identifying the cells expressing AGTR2 may clarify the likely role of AGTR2 in angiogenesis. AGTR2 expression has been associated with apoptosis in prostate cancer cells [35]. It needs to be determined if AGTR2 has a similar function in human bladder cancer cells. If it does have a similar effect, then reintroduction of this gene may eradicate tumors in the bladder.

Genes that were upregulated at day 7 represent early changes that have occurred in the bladder, while those that appear later may represent genes that promote survival of the tumor. Both MB49 and MBT-2 cells express the chemokine CXCL10 (IP10) as do human bladder tumors [36] and this chemokine has proinflammatory and angiostatic effects in the tumor microenvironment [37]. FOXP3-positive T cells and increased IL10 levels are present in human bladder cancers [38] and in the mice models. IL1b, IL6, GMCSF, MCSF, GCSF, and TNF*α* are expressed in a human bladder carcinoma cell line [39]. Local production of CCL2 (monocyte chemoattractant protein-1 (MCP-1)), in patients, correlated significantly with bladder cancer progression [40]. CCL22 (macrophage-derived chemokine (MDC)) and NOS2 have been shown to be expressed in clinical tissue specimens [36, 41]. Blocking CCL22 with siRNA during the differentiation and maturation of DC can block Treg recruitment [42], thus this is a good target for inhibition of tumor-induced immune suppression. IL13 suppression has both pro- and antitumor effects depending on the cell type producing it [22, 43]. A role for IL13 in human bladder cancer has not been determined as yet. PTGS2 or cyclooxygenase 2 (COX2) was found to be positive in 60% (368/617) of bladder cancer tissue [44]. In human urothelial cancer, TGF beta has been shown to correlate with disease progression [45]. Thus, the murine models are representative of the human disease and the new genes identified as being differentially expressed in this study may represent new targets for therapy of human bladder cancers.

Our data shows that there are both TH1 and TH2 genes upregulated by the presence of tumor cells. Arum et al. who evaluated a rat orthotopic model of bladder cancer also found that host immune response pathways were actively upregulated after tumor implantation [46]. Any cytokine that tilts the TH1/TH2 balance in favor of a TH1 response may induce tumor regression. It was recently reported that tilting the TH1/TH2 balance may block the immune suppression of CD8 T cells by mononuclear phagocytes [47, 48]. Better therapeutic responses could be induced by not only inducing TH1 responses but also concurrently suppressing TH2 responses. 

## Figures and Tables

**Table 1:** Primer sequences, annealing temperature, and fragment length of PCR products.

Gene	Primer sequence (5′–3′)	Temp. (°C)	Size (bp)
ACTB F	ACATGGAGAAGATCTGGCAC	58	660
ACTB R	CAGACAGCACTGTGTTGGCA		
CCL2 F	GCATCCACGTGTTGGCTCAG	60	383
CCL2 R	CACACTGGTCACTCCTACAG		
CCL22 F	CGTCCTTCTTGCTGTGGCAA	60	233
CCL22 R	CTTCTTCACCCAGACCTGCC		
CCL3 F	GCAACCAAGTCTTCTCAGCG	58	194
CCL3 R	CTTGGACCCAGGTCTCTTTG		
CCL5 F	GGTACCATGAAGATCTCTGC	51.5	286
CCL5 R	CTATCCTAGCTCATCTCC		
CCR2 F	GAGCCTGATCCTGCCTCTAC	58	371
CCR2 R	GGCACTGTTTGAAGAGACGT		
CD80 F	GCAGGATACACCACT	55	480
CD80 R	GGAAGCAAAGCAGG		
CSF2 F	TGGCCTGGGCTTCCTCAT	60	311
CSF2 R	GGATGACATGCCTGTCAC		
CXCL10 F	CGTGGTCACATCAGCTGCTA	58	244
CXCL10 R	TAGAACTGACGAGCCTGAGC		
CXCL11 F	AGGTCACAGCCATAGCCCTG	62	251
CXCL11 R	CCTGCATTATGAGGCGAGCTTGC		
FOXP3 F	TCGTAGCCACCAGTACTCAG	57	386
FOXP3 R	ATCTACGGTCCACACTGCTC		
GAPDH F	CACCCTGTTGCTGTAG	60	900
GAPDH R	GTCGGTGTGAACGGAT		
GATA3 F	GATAGCATGAAGCTGGAGACG	60	500
GATA3 R	AAGCTTGTAGTACAGCCCACA		
IFNg F	ACTGCCACGGCACAGTC	60	389
IFNg R	CCGCTTCCTGAGGCTG		
IL12a F	CCATCGATGAGCTGATGCAG	58	340
IL12a R	ATGCTGAGGTAGCTGTGCCA		
IL13 F	TGTCTCTCCCTCTGACCC	60	201
IL13 R	TACAGAGGCCATGCAATATCC		
IL15 F	ATGAACTGCTTTCTCCTGGAA	60	205
IL15 R	TGGACAATGCGTATAAAGCTTTGC		
IL17 F	CCAGGGAGAGCTTCATCTGT	58	431
IL17 R	AAGATGCTGGTGGGTGTGGG		
IL1b F	GGCAACTGTTCCTGAACTCAACTG	62	739
IL1b R	GCTTGTCTGCTGCTTGTGAGGTGC		
IL2 F	TGATGGACCTACAGGAGCTCCTGAG	60	191
IL2 R	GAGTCAAATCCAGAACATGCCGCAG		
IL6 F	GATGCAACCAAACTGGATATAATC	60	225
IL6 R	GAGCATTGGAAGTTGGGGTA		
NOS2 F	AAAGCCACGAGGCTCTGACA	58	259
NOS2 R	ACCATCACGCTCGAGGTTGA		
PTGS2 F	ATGCTCTTCCGAGCTGTGCT	55	239
PTGS2 R	GTGGGTCAGGATGTAGTGCA		
TBET F	CTAAAGCTCACCAACAACAAG	55	840
TBET R	GTTGGGAAAATAATTATAAAA		
TGFb1 F	CTGCAAGACCATCGACAT	55	580
TGFb1 R	ACAAGAGCAGTGAGCGCT		
TNF F	TGCACCACCATCAAGGACTC	58	360
TNF R	CAGCTCAGCTCCGTTTTCAC		

**Table 2:** Relative expression of genes in C57BL/6 mice bearing bladder tumors.

		Day 7		Day 28		
	Gene	Control (7)	Tumor (12)	Control (11)	Cured (4)	Tumor (15)
Realtime^†^	AGTR2	0.871 ± 0.123	0.231 ± 0.250*	1.381 ± 0.623	1.072 ± 0.251	0.171 ± 0.205*
	CSF2	0.944 ± 0.218	26.07 ± 28.00*	2.621 ± 5.554	1.637 ± 0.443	38.10 ± 37.28*
	CTLA4	1.088 ± 0.567	38.27 ± 36.06*	2.421 ± 6.216	3.026 ± 0.834	47.19 ± 30.92*
	GZMB	0.927 ± 0.312	27.07 ± 25.39*	0.966 ± 0.291	2.495 ± 0.456	7.349 ± 7.158*
	IFNg	1.178 ± 0.455	127.5 ± 127.9*	0.726 ± 0.542	2.005 ± 0.742	8.875 ± 10.89*
	IL10	5.022 ± 8.895	17.94 ± 36.01	1.377 ± 0.981	2.526 ± 0.975	3.749 ± 2.838*
	PTGS2	1.126 ± 0.781	2.424 ± 0.789*	1.245 ± 0.844	3.818 ± 3.132	3.040 ± 6.290
	TGFb1	0.934 ± 0.302	2.450 ± 2.500	1.251 ± 0.576	3.885 ± 3.301	2.110 ± 1.439
	TNF	0.956 ± 0.155	11.36 ± 12.66*	2.658 ± 5.417	3.333 ± 2.303	8.008 ± 14.67
	PSA	2.157 ± 1.898	126.7 ± 163.7*	0.491 ± 0.532	0.095 ± 0.099	243.4 ± 258.1*

RT-PCR	CCL2	1.140 ± 0.510	2.200 ± 0.840	0.799 ± 0.398	1.350 ± 0.616	1.996 ± 0.630*
	CCL22	0.063 ± 0.026	0.212 ± 0.062*	0.095 ± 0.019	0.139 ± 0.050	0.108 ± 0.070
	CCL3	0.193 ± 0.340	0.073 ± 0.044	0.928 ± 0.397	1.699 ± 2.210	2.366 ± 1.533
	CCL5	0.308 ± 0.206	0.613 ± 0.284	0.360 ± 0.050	0.670 ± 0.170	0.680 ± 0.090*
	CD80	0.094 ± 0.055	0.367 ± 0.094*	0.016 ± 0.008	0.134 ± 0.268	0.631 ± 0.825
	CXCL10	2.383 ± 1.339	591.9 ± 257.8*	1.854 ± 1.257	4.831 ± 2.943	17.04 ± 30.28
	CXCL11	0.523 ± 0.351	1.445 ± 0.374	0.297 ± 0.390	0.854 ± 0.656	2.240 ± 2.576*
	FOXP3	0.016 ± 0.007	0.217 ± 0.203	0.042 ± 0.018	0.068 ± 0.058	0.155 ± 0.141
	GATA3	0.901 ± 0.327	0.118 ± 0.039*	0.858 ± 0.173	0.935 ± 0.203	0.584 ± 0.244
	IL12a	0.014 ± 0.008	0.064 ± 0.031*	—	—	—
	IL13	0.020 ± 0.010	0.600 ± 0.220*	0.118 ± 0.277	0.060 ± 0.063	0.033 ± 0.013
	IL15	1.295 ± 0.685	0.773 ± 0.381	0.298 ± 0.149	0.218 ± 0.255	0.225 ± 0.189
	IL1b	0.125 ± 0.230	1.174 ± 0.578*	0.006 ± 0.003	0.011 ± 0.005	0.211 ± 0.177
	IL2	0.681 ± 0.714	0.994 ± 0.609	2.098 ± 1.635	1.764 ± 0.804	1.582 ± 1.329
	IL6	1.550 ± 0.670	3.190 ± 0.710*	0.829 ± 0.099	1.482 ± 1.026	4.957 ± 2.054*
	NOS2	0.580 ± 0.100	0.950 ± 0.230*	—	—	—
	TBET	0.057 ± 0.009	0.051 ± 0.013	0.083 ± 0.068	0.097 ± 0.068	0.075 ± 0.035

*Note*. The sample size for each group is indicated in brackets and “—” indicates not done. ^†^Data is presented as the mean RQ ± SD. *indicates *P* < 0.05 with respect to control.

**Table 3:** Immune cells present in the bladder at day 28.

Markers	Control	Tumor
CD3^+^CD4^+^	0.593 ± 0.577	1.387 ± 1.171
CD3^+^CD8a^+^	0.000 ± 0.000	2.460 ± 0.716*
CD4^+^CD25^+^	4.203 ± 4.076	1.810 ± 0.605
CD45R/B220^+^	0.043 ± 0.075	2.037 ± 0.926*
pan-NK^+^	7.003 ± 1.851	5.613 ± 2.470
Mac3^+^	9.670 ± 1.769	14.54 ± 6.187

*denotes *P* < 0.05 with respect to control.

**Table 4:** Bladder gene expression at day 28 segregated by PSA expression.

	Day 28				
Group	Control	Cured	Tumor (small)	Tumor (medium)	Tumor (large)
Gene/no	11	4	5	6	4
PSA	0.491 ± 0.532	0.095 ± 0.099	73.03 ± 69.92	311.2 ± 45.85	1602 ± 1914*
AGTR2	1.381 ± 0.623	1.072 ± 0.251	0.342 ± 0.249*	0.018 ± 0.011*	0.187 ± 0.130*
CSF2	2.621 ± 5.554	1.637 ± 0.443	18.16 ± 21.55	28.88 ± 12.69	76.86 ± 52.56*
CTLA4	2.421 ± 6.216	3.026 ± 0.834	16.42 ± 15.00	68.02 ± 26.53*	54.42 ± 21.72*
GZMB	0.966 ± 0.291	2.495 ± 0.456	8.394 ± 11.47*	5.305 ± 2.977	9.108 ± 5.886*
IFNg	0.726 ± 0.542	2.005 ± 0.742	10.54 ± 15.36	4.594 ± 3.200	13.22 ± 12.45*
IL10	1.377 ± 0.981	2.526 ± 0.975	5.493 ± 4.593*	3.301 ± 1.454	2.677 ± 2.072
PTGS2	1.245 ± 0.844	3.818 ± 3.132	1.877 ± 2.350	4.594 ± 10.00	2.163 ± 1.499
TGFb1	1.251 ± 0.576	3.885 ± 3.301	1.669 ± 1.321	1.826 ± 0.698	2.835 ± 2.072
TNF	2.658 ± 5.417	3.333 ± 2.303	3.148 ± 3.013	3.721 ± 1.815	20.51 ± 26.49*

Data is expressed as the mean RQ ± SD. *N*: number of mice samples analyzed; “—”: not done. “*” Significant (*P* < 0.05) with respect to control.

## References

[1] O'Donnell MA (2009). Optimizing BCG therapy. *Urologic Oncology*.

[2] Herr HW, Morales A (2008). History of bacillus calmette-guerin and bladder cancer: an immunotherapy success story. *Journal of Urology*.

[3] Chen F, Zhang G, Cao Y, Hessner MJ, See WA (2009). MB49 murine urothelial carcinoma: molecular and phenotypic comparison to human cell Llines as a model of the direct tumor response to bacillus calmette-guerin. *Journal of Urology*.

[4] Horiguchi Y, Larchian WA, Kaplinsky R, Fair WR, Heston WDW (2000). Intravesical liposome-mediated interleukin-2 gene therapy in orthotopic murine bladder cancer model. *Gene Therapy*.

[5] Shiau AL, Lin CY, Tzai TS, Wu CL (2001). Postoperative immuno-gene therapy of murine bladder tumor by in vivo administration of retroviruses expressing mouse interferon-*γ*. *Cancer Gene Therapy*.

[6] Zang Z, Mahendran R, Wu Q, Yong T, Esuvaranathan K (2004). Non-viral tumor necrosis factor-alpha gene transfer decreases the incidence of orthotopic bladder tumors. *International Journal of Molecular Medicine*.

[7] Wu Q, Mahendran R, Esuvaranathan K (2003). Nonviral cytokine gene therapy on an orthotopic bladder cancer model. *Clinical Cancer Research*.

[8] Wu Q, Esuvaranathan K, Mahendran R (2004). Monitoring the response of orthotopic bladder tumors to granulocyte macrophage colony-stimulating factor therapy using the prostate-specific antigen gene as a reporter. *Clinical Cancer Research*.

[9] Chen L, Chen D, Block E, O’Donnell M, Kufe DW, Clinton SK (1997). Eradication of murine bladder carcinoma by intratumor injection of a bicistronic adenoviral vector carrying cDNAs for the IL-12 heterodimer and its inhibition by the IL-12 p40 subunit homodimer. *The Journal of Immunology*.

[10] Loskog ASI, Fransson ME, Totterman TTH (2005). AdCD40L gene therapy counteracts T regulatory cells and cures aggressive tumors in an orthotopic bladder cancer model. *Clinical Cancer Research*.

[11] Hikosaka S, Hara I, Miyake H, Hara S, Kamidono S (2004). Antitumor effect of simultaneous transfer of interleukin-12 and interleukin-18 genes and its mechanism in a mouse bladder cancer model. *International Journal of Urology*.

[12] Buscarini M, Quek ML, Gilliam-Hegarich S, Kasahara N, Bochner B (2007). Adenoviral receptor expression of normal bladder and transitional cell carcinoma of the bladder. *Urologia Internationalis*.

[13] Lawrencia C, Mahendran R, Esuvaranathan K (2001). Transfection of urothelial cells using methyl-*β*-cyclodextrin solubilized cholesterol and Dotap. *Gene Therapy*.

[14] Harimoto K, Sugimura K, Lee CR, Kuratsukuri K, Kishimoto T (1998). In vivo gene transfer methods in the bladder without viral vectors. *British Journal of Urology*.

[15] Yang AS, Lattime EC (2003). Tumor-induced interleukin 10 suppresses the ability of splenic Dendritic Cells to stimulate CD4 and CD8 T-cell responses. *Cancer Research*.

[16] Halak BK, Maguire HC, Lattime EC (1999). Tumor-induced interleukin-10 inhibits type 1 immune responses directed at a tumor antigen as well as a non-tumor antigen present at the tumor site. *Cancer Research*.

[17] Ninalga C, Loskog A, Klevenfeldt M, Essand M, Tötterman TH (2005). CpG oligonucleotide therapy cures subcutaneous and orthotopic tumors and evokes protective immunity in murine bladder cancer. *Journal of Immunotherapy*.

[18] Cai JH, Deng S, Kumpf SW (2007). Validation of rat reference genes for improved quantitative gene expression analysis using low density arrays. *BioTechniques*.

[19] Seow SW, Rahmat JN, Bay BH, Lee YK, Mahendran R (2008). Expression of chemokine/cytokine genes and immune cell recruitment following the instillation of Mycobacterium bovis, bacillus Calmette- Guérin or Lactobacillus rhamnosus strain GG in the healthy murine bladder. *Immunology*.

[20] Summerhayes IC, Franks LM (1979). Effects of donor age on neoplastic transformation of adult mouse bladder epithelium in vitro. *Journal of the National Cancer Institute*.

[21] Loskog A, Ninalga C, Hedlund T, Alimohammadi M, Malmström PU, Tötterman TH (2005). Optimization of the MB49 mouse bladder cancer model for adenoviral gene therapy. *Laboratory Animals*.

[22] Deepak P, Kumar S, Acharya A (2008). Gender variation in interleukin-13 production: a possible mechanism of differential in vivo growth of a T-cell lymphoma. *Scandinavian Journal of Immunology*.

[23] Wang X, Colby JKL, Rengel RC, Fischer SM, Clinton SK, Klein RD (2009). Overexpression of cyclooxygenase-2 (COX-2) in the mouse urinary bladder induces he expression of Immune- And cell proliferation-related genes. *Molecular Carcinogenesis*.

[24] Liakou CI, Kamat A, Tang DN (2008). CTLA-4 blockade increases IFN*γ*-producing CD4+ICOS hi cells to shift the ratio of effector to regulatory T cells in cancer patients. *Proceedings of the National Academy of Sciences of the United States of America*.

[25] Sandes EO, Faletti AG, Riveros MD (2005). Expression of inducible nitric oxide synthase in tumoral and non-tumoral epithelia from bladder cancer patients. *Nitric Oxidey*.

[26] Marhaba R, Nazarenko I, Knöfler D (2008). Opposing effects of fibrosarcoma cell-derived IL-1*α* and IL-1*β* on immune response induction. *International Journal of Cancer*.

[27] Qin XJ, Shi HZ, Deng JM, Liang QL, Jiang J, Ye ZJ (2009). CCL22 recruits CD4-positive CD25-positive regulatory T cells into malignant pleural effusion. *Clinical Cancer Research*.

[28] Obiri NI, Husain SR, Debinski W, Puri RK (1996). Interleukin 13 inhibits growth of human renal cell carcinoma cells ependently of the p140 interleukin 4 receptor chain. *Clinical Cancer Research*.

[29] Sharma P, Shen Y, Wen S (2007). CD8 tumor-infiltrating lymphocytes are predictive of survival in muscle-invasive urothelial carcinoma. *Proceedings of the National Academy of Sciences of the United States of America*.

[30] Seow SW, Cai S, Rahmat JN (2010). Lactobacillus rhamnosus GG induces tumor regression in mice bearing orthotopic bladder tumors. *Cancer Science*.

[31] Sharma P, Shen Y, Wen S (2006). Cancer-testis antigens: expression and correlation with survival in human urothelial carcinoma. *Clinical Cancer Research*.

[32] Chow L, Rezmann L, Imamura K (2008). Functional angiotensin II type 2 receptors inhibit growth factor signaling in LNCaP and PC3 prostate cancer cell lines. *Prostate*.

[33] Kang KH, Park SY, Rho SB, Lee JH (2008). Tissue inhibitor of metalloproteinases-3 interacts with angiotensin II type 2 receptor and additively inhibits angiogenesis. *Cardiovascular Research*.

[34] Walther T, Menrad A, Orzechowski HD, Siemeister G, Paul M, Schirner M (2003). Differential regulation of in vivo angiogenesis by angiotensin II receptors. *The FASEB Journal*.

[35] Li H, Qi Y, Li C (2009). Angiotensin type 2 receptor-mediated apoptosis of human prostate cancer cells. *Molecular Cancer Therapeutics*.

[36] Yamada H, Luo Y, Matsumoto T, O’Donnell MA (2005). A novel expression of macrophage derived chemokine in human bladder cancer. *Journal of Urology*.

[37] Yang J, Richmond A (2004). The angiostatic activity of interferon-inducible protein-10/CXCL10 in human melanoma depends on binding to CXCR3 but not to glycosaminoglycan. *Molecular Therapy*.

[38] Loskog A, Ninalga C, Paul-Wetterberg G, de la Torre M, Malmström PU, Tötterman TH (2007). Human bladder carcinoma is dominated by T-regulatory cells and Th1 inhibitory cytokines. *Journal of Urology*.

[39] Kaashoek JGJ, Mout R, Falkenburg JHF, Willemze R, Fibbe WE, Landegent JE (1991). Cytokine production by the bladder carcinoma cell line 5637: rapid analysis of mRNA expression levels using a cDNA-PCR procedure. *Lymphokine and Cytokine Research*.

[40] Amann B, Perabo FGE, Wirger A, Hugenschmidt H, Schultze-Seemann W (1998). Urinary levels of monocyte chemo-attractant protein-1 correlate with tumour stage and grade in patients with bladder cancer. *British Journal of Urology*.

[41] Wolf H, Haeckel C, Roessner A (2000). Inducible nitric oxide synthase expression in human urinary bladder cancer. *Virchows Archiv*.

[42] Kang S, Xie J, Ma S, Liao W, Zhang J, Luo R (2010). Targeted knock down of CCL22 and CCL17 by siRNA during DC differentiation and maturation affects the recruitment of T subsets. *Immunobiology*.

[43] Park JM, Terabe M, Donaldson DD, Forni G, Berzofsky JA (2008). Natural immunosurveillance against spontaneous, autochthonous breast cancers revealed and enhanced by blockade of IL-13-mediated negative regulation. *Cancer Immunology, Immunotherapy*.

[44] Wild PJ, Kunz-Schughart LA, Stoehr R (2005). High-throughput tissue microarray analysis of COX2 expression in urinary bladder cancer. *International Journal of Oncology*.

[45] Hung TT, Wang H, Kingsley EA, Risbridger GP, Russell PJ (2008). Molecular profiling of bladder cancer: involvement of the TGF-*β* pathway in bladder cancer progression. *Cancer Letters*.

[46] Arum CJ, Anderssen E, Viset T (2010). Cancer immunoediting from immunosurveillance to tumor escape in microvillus-formed niche: a study of syngeneic orthotopic rat bladder cancer model in comparison with human bladder cancer. *Neoplasia*.

[47] Adam L, Black PC, Kassouf W (2007). Adenoviral mediated interferon-*α* 2b gene therapy suppresses the pro-angiogenic effect of vascular endothelial growth factor in superficial bladder cancer. *Journal of Urology*.

[48] Nonaka K, Saio M, Suwa T (2008). Skewing the Th cell phenotype toward Th1 alters the maturation of tumor-infiltrating mononuclear phagocytes. *Journal of Leukocyte Biology*.

